# Process optimization to produce anisotropic NdFeB permanent magnets from recycled powder via powder extrusion

**DOI:** 10.1038/s41598-025-19111-6

**Published:** 2025-10-09

**Authors:** Stefan Rathfelder, Stephan Schuschnigg, Christian Kukla, Clemens Holzer, Dieter Suess, Carlo Burkhardt

**Affiliations:** 1 Institute for Precious and Technology Metals, Pforzheim University, Tiefenbronner Str. 65, 75175 Pforzheim, Germany; 2 Department of Polymer Engineering and Science, Institute of Polymer Processing, Otto Gloeckel-Straße 2, Montanuniversitaet Leoben, 8700 Austria; 3 Research and Innovation Service, Franz-Josef-Strasse 18, Montanuniversitaet Leoben, 8700 Austria; 4 Faculty of Physics, University of Vienna, Boltzmanngasse 1, Vienna, 1090 Austria

**Keywords:** Powder extrusion moulding (PEM), Metal injection moulding (MIM), Anisotropic NdFeB permanent magnets, Recycling of eol-magnets, Engineering, Materials science

## Abstract

In this study, process parameters are presented on how to optimize the novel process for producing anisotropic NdFeB permanent magnets from recycled materials using powder extrusion moulding (PEM). The process is based on the recycling of end-of-life (Eol) magnets that have been processed into powder by the Hydrogen Processing of Magnetic Scrap (HPMS) process. In this study, the influence of melt temperature on the alignment behaviour of magnetic particles during in situ alignment using the PEM process is investigated. This process enabled the fabrication of sintered, anisotropic NdFeB permanent magnets with a high degree of alignment, as indicated by a *B*_*r*_*/J*_*s*_ ratio of 0.96. Moreover, the challenges associated with the production of anisotropic NdFeB permanent magnets with PEM are delineated in this study. The primary focus of this study is on the carbon and oxygen content. The study found that sintered magnets with a carbon content of approximately 0.045 wt% could be produced using the PEM process. The oxygen content, however, exhibited a variation depending on the initial material used. Nonetheless, the oxygen content increased by approximately 0.4 wt% from the HPMS powder to the sintered part.

**Supplementary Information:**

The online version contains supplementary material available at 10.1038/s41598-025-19111-6.

## Introduction

Rare earth elements (REEs) are indispensable for European industries, particularly for the production of permanent magnets utilised in wind turbines and electric mobility. Wind energy represents one of the most cost-effective and rapidly growing technologies for ecological transition, with a significant reliance on permanent magnet synchronous generators (PMSGs) in offshore applications due to their compact size and efficiency. The European Raw Materials Alliance (ERMA) projects a significant increase in offshore wind power capacity from 12 GW to 73 GW by 2030, necessitating the production of approximately 40,000 tonnes of magnetic material for the additional 61 GW^1^. Furthermore, permanent magnets are integral to electric motors utilized in mobility applications, including electric vehicles (EVs), trains, and heavy transport. The transition to electric drives is driving an exponential rise in demand for NdFeB magnets, which is forecast to increase from 5,000 tonnes annually in 2019 to 70,000 tonnes by 2030. As 95% of electric vehicles (EVs) rely on rare-earth permanent magnets (REPMs) as of 2022, this shift underscores their importance in achieving sustainable Mobility. In addition to these sectors, REPMs are essential for industrial and service robotics, with an industrial robot requiring approximately 15 kg of magnets for its drives. Further applications include hard disks, heat pumps, and drones^2^. The dominance of China in mining REEs and Europe’s reliance on imports demonstrate the urgent need for sustainable and resilient supply chains to meet the growing demand. One way to reduce this dependency is by recycling magnets already in use in Europe, ensuring a more resilient and independent supply chain. The SUSMAGPRO and REESilience projects were focused on developing efficient recycling processes for rare earth magnets, improving resource sustainability and security of supply^3,4^.

The Powder Extrusion Moulding (PEM) process represents a promising approach to produce near-net-shape isotropic^5^ and anisotropic permanent magnets^5,6^. PEM is a cost-effective manufacturing process because it eliminates the need for expensive post-processing steps, such as wire cutting or grinding, which are commonly required for sintered NdFeB magnets produced via the conventional powder metallurgy route. The PEM process is closely related to the Metal Injection Moulding (MIM) process, and both share a similar sequence of processing steps. These processes typically comprise four main stages: feedstock preparation, shaping, debinding, and sintering. The only difference between the two processes lies in the shaping stage, which is performed by injection molding in the case of MIM and by extrusion in the case of PEM.

Several studies have demonstrated the feasibility of producing NdFeB permanent magnets from both virgin and recycled material using the Metal Injection Moulding (MIM) process.

Kukla et al.^7^ successfully fabricated isotropic NdFeB permanent magnets from recycled material using the MIM process. Their study demonstrated that by selectively adjusting the heating rate during thermal debinding and optimizing the sintering temperature, recycled NdFeB magnets can achieve magnetic properties comparable to those of magnets produced from virgin material. Burkhardt et al.^8^ also demonstrated in their study the feasibility of producing NdFeB permanent magnets from recycled material. Isotropic NdFeB magnets were successfully fabricated using the MIM process. The powder employed was derived from an end-of-life (Eol) magnet and processed via Hydrogen Processing of Magnetic Scrap (HPMS).

In addition, Hartwig et al.^9^ reported the successful production of anisotropic NdFeB permanent magnets from virgin material via MIM. Their work focused on the selection of suitable binder systems and demonstrated that low heating rates during thermal debinding contribute positively to reducing residual carbon content. Furthermore, they analysed the impact of different powder loadings on the alignment of magnetic particles and on the shrinkage behaviour of the moulded parts during sintering.

In both the MIM and PEM processes, the powder is mixed with a binder system composed of polymers, waxes, and various additives to form a homogeneous mixture known as feedstock. Binder systems used in NdFeB feedstocks for MIM typically consist of multiple components, including a main binder, a backbone binder, and functional additives to facilitate processing and improve performance Recent developments, as described in the review article by Crozier–Bioud et al. ^10^, have highlighted polyethylene (PE) as a widely used backbone binder.

A key challenge in the production of anisotropic NdFeB permanent magnets using MIM and PEM processes is the implementation of magnetic pre-alignment of the magnetic moments. In both processes, an external magnetic field is applied that must be sufficiently strong to overcome the resistance of the binder matrix to the rotation of the magnetic particles. In the MIM process, this is typically achieved by integrating a magnetization unit into the injection mould, allowing the alignment of magnetic particles during or immediately after shaping, while the binder is still in a low-viscosity state^11^. The alignment of magnetic particles is achieved through the application of a magnetic field prior to the solidification of the melt. In the PEM process, achieving particle alignment is more challenging, as the extruded strand continuously flows through an external magnetic field during shaping. Due to the continuous movement in PEM, a constant magnetic field is required to achieve alignment throughout the extrusion process. The alignment methodology and appropriate tooling for this purpose have been described in detail in another publication^6^.

Various factors influence the alignment of magnetic particles during MIM or PEM. The orientation model by Jung et al.^12^ describes the balance between magnetic torque, hydrodynamic torque, and particle–particle interactions, and introduces the dimensionless Mason number, as a measure of the ratio between hydrodynamic and magnetic torques, Eq. 1.

1 $$\:\text{M}\text{a}=\frac{{\upeta\:}\cdot\:{\upgamma\:}\dot{}}{{H}_{r}*{H}_{0}},$$

Here, η is the viscosity of the binder matrix, γ˙ the shear rate, H_0_ the applied magnetic field strength, and H_r_ a characteristic field derived from the magnetic properties of the particles. Since the viscosity is temperature dependent^11^, variations in processing temperature directly influence the Mason number and thus the expected particle alignment. A high Mason number (Ma≫1) indicates that hydrodynamic forces dominate, making magnetic alignment difficult, whereas a low Mason number (Ma≪1) means that magnetic torque overcomes viscous drag. Since the viscosity is temperature dependent, variations in processing temperature directly influence the Mason number and thus the expected particle alignment.

Sarkar et al.^13^ experimentally demonstrated with an extrusion-based 3D printing process that the degree of particle alignment depends on both the temperature and the applied magnetic field. The researchers explain that as the nozzle temperature increases, the viscosity of the binder decreases, which allows the magnetic particles to rotate and align more effectively in the direction of the applied field. By optimizing the melting temperature, the degree of particle alignment can be significantly improved, leading to enhanced remanence *B*_*r*_ and a higher maximum energy product *BH*_max_.

Another major challenge in the production of NdFeB permanent magnets using a powder-binder-based process is the prevention of undesirable impurities within the microstructure, particularly carbon and oxygen. These impurities predominantly react with the Nd-rich phase located at the grain boundaries^14^.

Debinding is one of the most critical processing steps, as it has a direct impact on the residual carbon content within the microstructure. To achieve a fully metallic structure during sintering, the binder system must be thoroughly removed beforehand. Incomplete removal can leave behind residual carbon, which negatively affects both the microstructure and the magnetic performance of the final magnet. Specifically, carbon can react with the Nd-rich grain boundary phase, leading to the formation of carbides and α–Fe. These secondary phases compromise the magnetic properties, most notably by reducing the coercivity *H*_*cj*_^14–17^.

The debinding strategy must be tailored to the specific binder system employed. In the case of NdFeB green parts produced via MIM, usually a multi-component binder system consisting of paraffin wax, polymer waxes, and a polyethylene (PE) backbone binder is used. Consequently, a two-stage debinding approach is applied, typically involving an initial solvent debinding step to remove the base binder components, followed by thermal debinding to eliminate the remaining polymeric backbone binder.

Momeni et al.^18^ successfully fabricated MIM components using a feedstock composed of NdFeB powder and a multi-component binder system containing Linear Low Density Polyethylene (LLDPE) as the backbone, paraffin waxes, and stearic acid. Solvent debinding was performed in cyclohexane at 40 °C for 12 h to remove the paraffin wax and likely a portion of the stearic acid. This was followed by thermal debinding in a hydrogen atmosphere, applying a controlled heating rate of 1 K/min with four temperature plateaus at 250 °C, 300 °C, 420 °C, and 620 °C, each held for four hours. Using this method, NdFeB magnets with carbon contents between 1165 ppm and 2012 ppm were obtained.

The influence of heating rates and atmospheric conditions during thermal debinding was investigated by Lopes et al.^19^, who studied the debinding behaviour of a feedstock composed of PE, wax, and stearic acid. In the first stage, the primary binder components were removed by solvent debinding using hexane at room temperature for 48 h. This was followed by thermal debinding carried out in both argon and hydrogen atmospheres at heating rates of 0.5 °C/min, 1.0 °C/min, and 1.5 °C/min. The study demonstrated that low heating rates in combination with a hydrogen atmosphere resulted in significantly reduced residual carbon content. At slower heating rates, the binder undergoes more complete decomposition, an effect further enhanced by the presence of hydrogen, which reacts with the decomposing polymer.

Subsequently, the debinded parts, designated as brown parts, undergo sintering in a sintering furnace to yield a metallic structure. It is imperative that sintering is carried out in vacuum or inert atmosphere to prevent the Nd-rich phase from oxidising. In the production of sintered NdFeB permanent magnets, the absorption of oxygen during processing is unavoidable. Oxygen uptake during powder preparation and sintering results in the formation of neodymium oxide (Nd_2_O_2_), which depletes the amount of Nd available for the formation of the Nd_2_Fe_14_B magnetic phase. Consequently, the formation of the desired Nd_2_Fe_14_B phase is suppressed, and the excess iron, no longer incorporated into the magnetic structure, precipitates as soft magnetic α–Fe^20^.

Elevated oxygen levels impair the wettability of the Nd-rich phase, which compromises the effective isolation of the Nd_2_Fe_14_B grains. This results in the formation of grain boundary precipitates and a disrupted microstructural interface. Consequently, the magnetic properties, particularly the coercivity, are significantly degraded^21^.

It is of particular importance to monitor the process steps and avoid these impurities, especially when working with recycled materials. As these materials already have somewhat higher oxygen and carbon values than new materials, the process window for the production of magnets with favourable magnetic properties is therefore smaller^8^.

In this study, extrusion trials were conducted at various temperatures to determine the optimal processing temperature at which the magnetic particles achieve the highest degree of alignment. These experiments were performed using starting materials derived from two different Eol magnets. In addition, the overall process was optimized to enable the production of anisotropic NdFeB permanent magnets from recycled material with low oxygen and carbon content, ensuring that the magnetic properties are not adversely affected.

## Experimental

### Process description

**Fig. 1:**
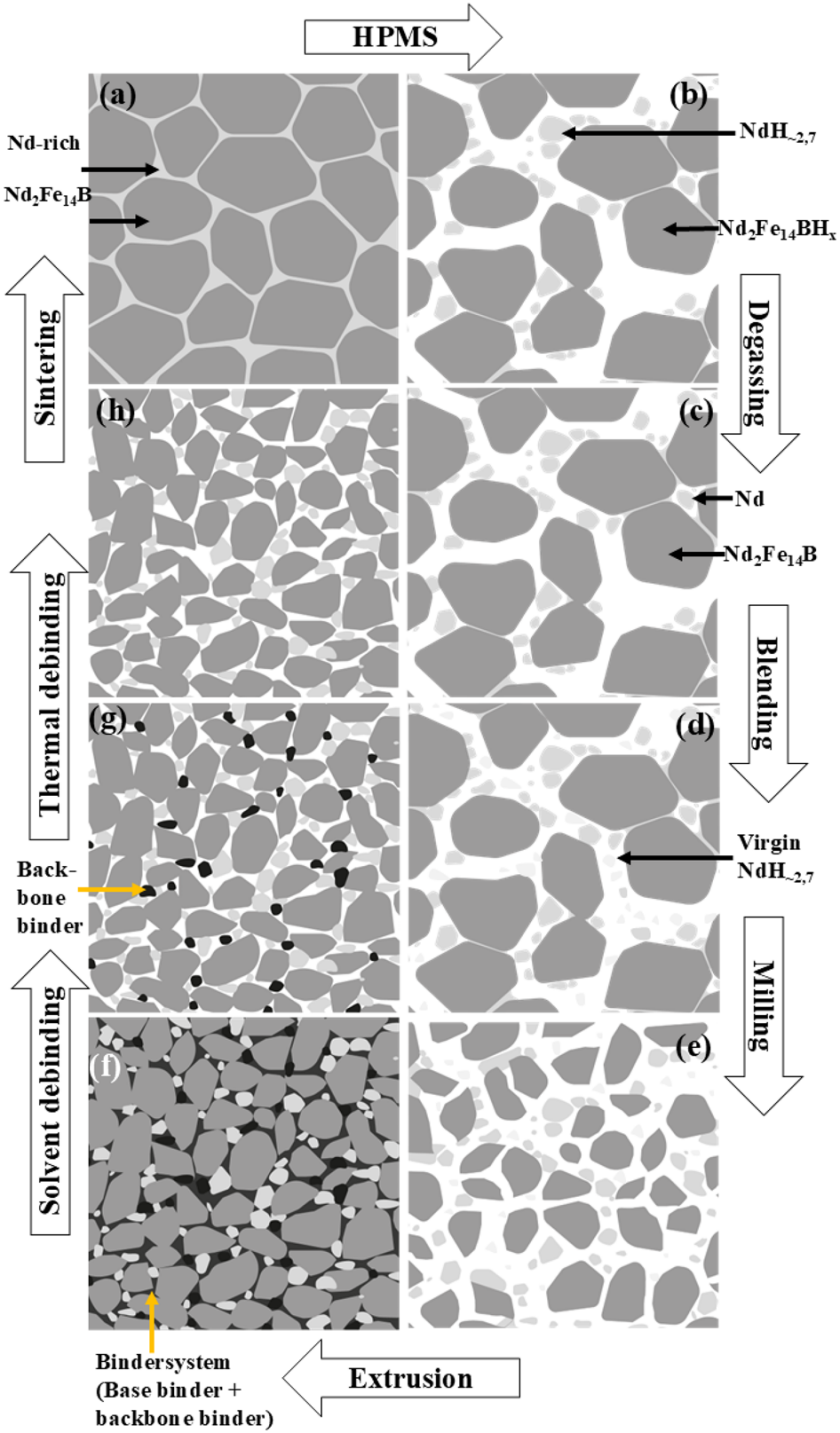
Process steps for the production of anisotropic, sintered PEM magnets from HPMS powder, according to^5^.

The process flow for the production of anisotropic NdFeB permanent magnets from recycled material is illustrated in Fig. 1. The individual process steps are explained in greater detail below. The manufacturing process encompasses all stages up to the completion of the final anisotropic PEM magnet.

In this study, two distinct end-of-life (Eol) magnets were used as starting materials. Magnet M1 was obtained from a decommissioned wind turbine, whereas the origin of magnet M2 could not be identified. Two kilograms of magnetic scrap from each magnet were processed into powder.

To prevent contamination by coating residues, the protective coatings were removed prior to powder processing using sandblasting. According to Grau et al.^22^, sandblasting is a common method for removing coatings and surface impurities from the NdFeB magnets. Both M1 and M2 were coated with epoxy. During sandblasting, the epoxy layer simply breaks away, making it easy to visually confirm whether the coating has been completely removed (Supplementary Figure A1).

The two starting magnets were processed into powder using the Hydrogen Processing of Magnetic Scrap (HPMS) method^23–25^.

HPMS involves exposing Eol magnets to a hydrogen atmosphere inside a sealed reactor. Hydrogen reacts with the Nd-rich phase, causing volumetric expansion and fracturing the sintered magnets into coarse powder. This process results in the formation of two phases: Nd_2_Fe_14_BH_x_ and NdH_~2.7_. The typical particle size of the Nd_2_Fe_14_BH_x_ phase is 10 μm, while NdH_~2.7_. particles are typically below 1 μm, as illustrated schematically in Fig. 1b.

To prevent oxidation, the HPMS process was conducted under an argon atmosphere in a glove box. During hydrogenation, the reactor pressure was maintained at 3 bar until the reaction was complete. The introduction of hydrogen during the HPMS process significantly reduces the material’s anisotropy field^26,27^. Consequently, a degassing process is necessary to remove the hydrogen and restore the material’s magnetic properties. It has been shown that hydrogen can be effectively removed from the powder by degassing at 600 °C for 2 h under vacuum^27,28^. Therefore, in this study, the HPMS powder was degassed under these conditions.

Prior to milling, 3 wt% NdH_~2.7_ was added to the degassed HPMS powder to compensate for the typically higher oxygen content in recycled materials compared to virgin alloys^8^, Fig. 1d. The powder blend was milled using a Retsch NANO 500 MM vibratory mill (Retsch GmbH, Haan, Germany) in three cycles at 50 Hz for 10 min each.

The feedstock is composed of three key components: a main binder, a backbone binder, and stearic acid. The main binder, consisting of paraffin waxes and polymer waxes, is prepared under an argon atmosphere to minimize oxidation of the sensitive NdFeB powder. This binder serves a dual purpose: it provides the necessary flowability for extrusion processing and simultaneously forms a protective coating around the powder particles to inhibit oxidation. Stearic acid is added to the formulation to improve the wettability between the binder system and the powder, thereby enhancing dispersion and homogeneity. The binder system used in the present study is based on the formulation from Gonzales–Gutierrez et al.^29^. In the production of the feedstock, the wax components were initially dissolved in an organic solvent under constant stirring. Subsequently, the milled NdFeB and NdH_~2.7_ powders and the stearic acid were added to the dissolved binder solution and thoroughly mixed to form a homogeneous blend. The resulting mixture was then dried under vacuum for 24 h to ensure complete removal of the solvent. The dried, coated powder was subsequently crushed to produce granules suitable for further processing in the extrusion system. After drying and crushing, the granulated, binder-coated powder was further processed in a co-rotating twin-screw extruder (KETSE 12/36, Anton Paar TorqueTec GmbH, Duisburg, Germany) to incorporate the thermoplastic backbone binder, polyethylene (PE). The twin-screw configuration ensures thorough distributive and dispersive mixing, enabling homogeneous integration of all binder components into the powder matrix and resulting in a uniform, extrusion-ready feedstock^5^.

**Fig. 2:**
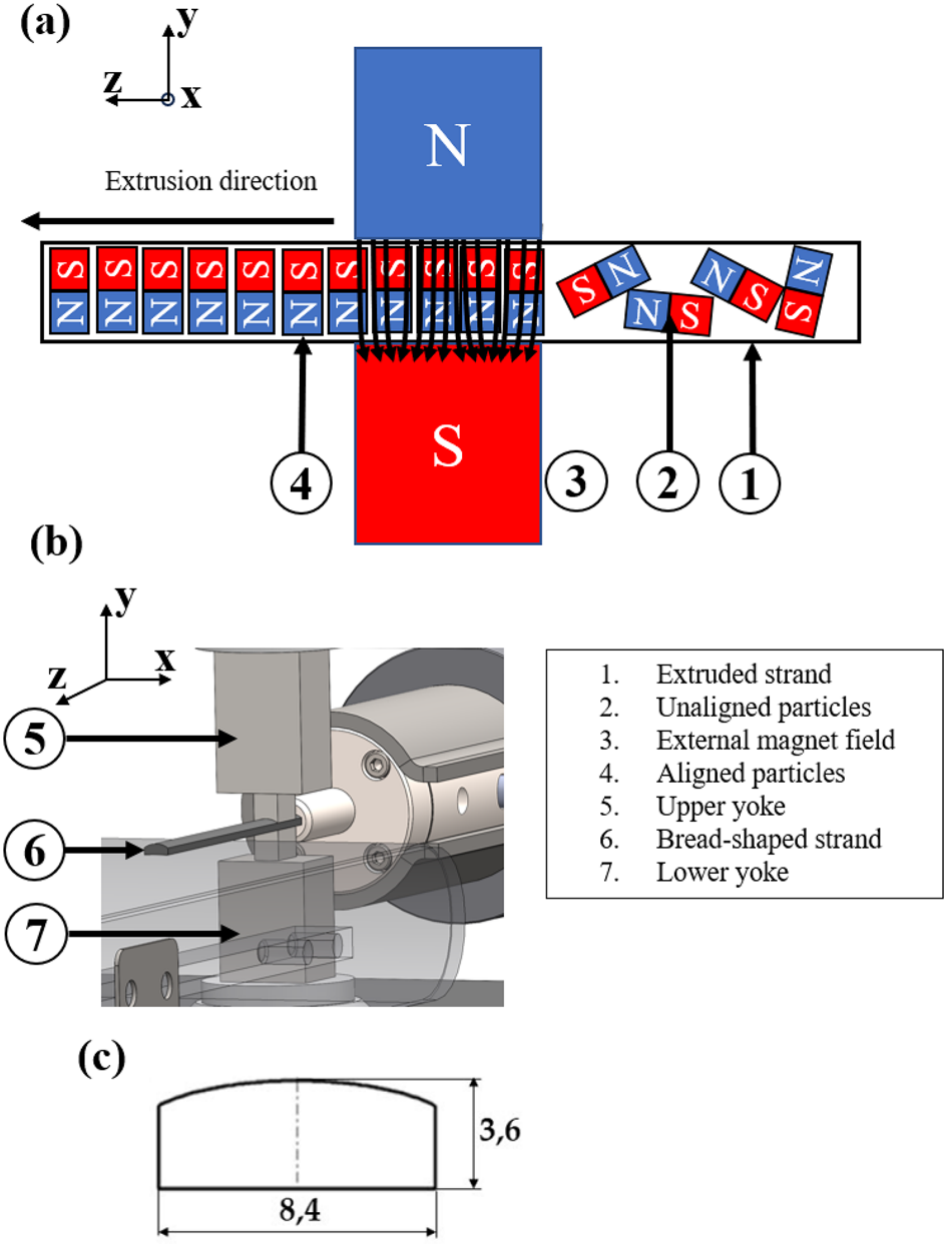
Schematic representation of the alignment process of the magnetic particles according to^6^.

A schematic representation of the extrusion and alignment process is shown in Fig. 2a. The formed strand (1) of NdFeB feedstock and the unaligned NdFeB particles (2) are passing through an external magnetic field generated by an electromagnet (3). The application of this magnetic field results in the alignment of the magnetic particles, with their orientation becoming parallel (4) to the magnetic field. Figure 2b presents the 3D CAD model of the extrusion die, alignment tool, and extruded strand (6). Figure 2c shows the bread-shaped cross-section of the extruded strand, a geometry that is often used for magnets in electric motor applications.

To ensure effective alignment of the magnetic particles, the upper yoke (5) and lower yoke (7) are designed with a stepped geometry that focuses the magnetic field precisely on the still-molten strand during extrusion The influence of the magnetic field and the geometry of the yoke on the in situ alignment of magnetic particles during extrusion has been described in^6^. The external magnetic field required for aligning the magnetized particles during extrusion was generated using the EM2000 electromagnet from Dr. Brockhaus Messtechnik GmbH & Co. KG (Lüdenscheid, Germany). In continuous operation, the maximum magnetic field was achieved at a current of 10 A. While higher currents could be applied for short periods, they caused significant heating of the device, making their use impossible for sustained operation. Due to the rounded geometry of the upper yoke and the resulting air gap, direct measurement of the magnetic flux density in the alignment zone using a Hall probe or Gaussmeter^11^ is susceptible to positioning errors. Therefore, the magnetic field distribution was determined by finite element method (FEM) simulation, as reported in a previous publication^6^. For the test setup used in the present study, the magnetic flux density obtained from the simulation was 1.35 T.

The powder content is a critical parameter in the PEM process: a low powder content can cause excessive shrinkage and deformation during sintering, whereas a high powder content can lead to void formation that persists after sintering. Extrusion tests with the binder system used in this study indicated that a powder content between 50 and 60 vol% is suitable for processing^5,29^. In the present work, all samples were produced with a powder loading of 50 vol%. This value had previously been used in alignment tests^6^, where it enabled the successful production of anisotropic NdFeB magnets. For this reason, the same powder loading was adopted in the present study. All extrusion trials were conducted using the starting materials M1 and M2, with the corresponding process parameters summarized in Table 1.

As explained by the orientation model of Jung et al.^12^, the viscosity of the binder influences the alignment of magnetic particles. Since binder viscosity is temperature dependent, extrusion and alignment experiments were carried out at five different nozzle temperatures (Table 1).

Before debinding and sintering, rectangular samples measuring 5 mm × 5 mm × 3 mm are cut from the green part. This ensures a flat and well-defined contact surface between the pole shoes when measuring the magnetic properties using the hystograph.

**Table 1:** Extrusion parameters with different temperature profiles.

Sample	Nozzle temp. [°C]	Starting material	Powder Loading	Amperage [A]
M1–T160°C	160	M1	50	10
M1–T170°C	170	M1	50	10
M1–T180°C	180	M1	50	10
M1–T190°C	190	M1	50	10
M1–T200°C	200	M1	50	10
M2–T160°C	160	M2	50	10
M2–T170°C	170	M2	50	10
M2–T180°C	180	M2	50	10
M2–T190°C	190	M2	50	10
M2–T200°C	200	M2	50	10

Following the shaping process, the polymer binder must be removed. For multi-component binder systems based on polymer waxes and thermoplastics, a two-stage debinding process is typically employed. In the first step, the main binder was removed with a solvent, followed by removal of the backbone binder by thermal debinding.

In this study, the main binder of the green parts was removed in cyclohexane heated to 60 °C^5,29^. The samples were fully immersed in the solvent for 24 h and the glass vessel was purged with argon after sealing to prevent oxidation of the solvent.

**Fig. 3:**
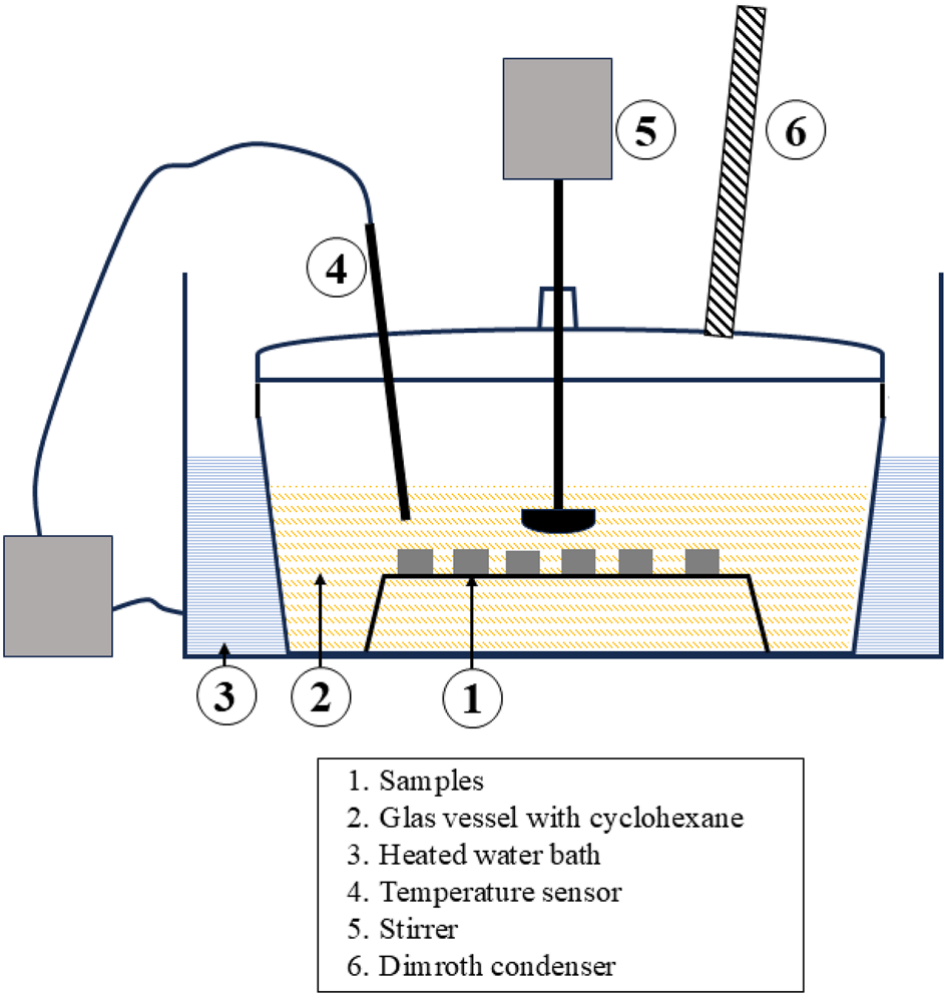
Laboratory setup of the solvent debinding station.

The experimental setup for the solvent debinding process is shown in Fig. 3. The glass container (1) holding the solvent is placed within a water-heated bath (2), with a temperature sensor (3) employed to monitor and log the temperature of the cyclohexane throughout the process. A stirring device (4) makes sure that the solvent is always being stirred. To minimize solvent loss, a Dimroth condenser (5) is used, which condenses vaporized cyclohexane via an internal spiral cooling coil. The condensed solvent then returns to the reaction vessel, enabling continuous operation without significant solvent evaporation.

Following solvent debinding, the samples exhibit an open-pored structure, rendering them highly susceptible to oxidation. To prevent oxidation during transfer from the debinding station to the sintering furnace, the samples were placed in ceramic crucibles filled with cyclohexane, which served as a protective medium. Upon placement in the furnace, the system was evacuated for 20 min, allowing the cyclohexane to evaporate prior to the onset of the sintering process.

**Fig. 4:**
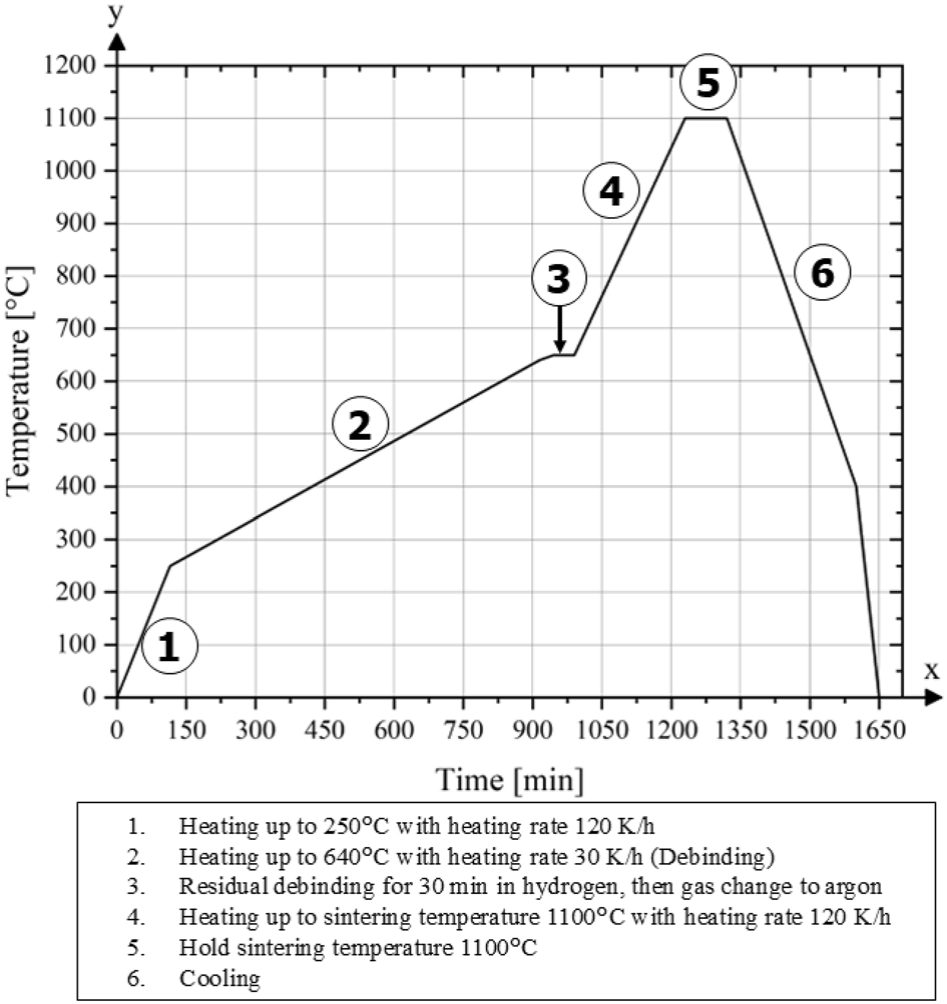
Thermal debinding and sintering curve.

Figure 4 presents the temperature–time profile applied during thermal debinding and subsequent sintering.

The thermal debinding and sintering process was carried out using a hinged furnace (FST 12/60/500, Carbolite Gero GmbH & Co. KG, Neuhausen, Germany), which is specifically designed for operation under hydrogen atmosphere at elevated pressures.

Thermogravimetric analysis (TGA), performed under an argon atmosphere using a Netzsch STA 449 C system (NETZSCH–Gerätebau GmbH, Selb, Germany), revealed that the decomposition of the backbone binder begins at approximately 290 °C. Consequently, a higher heating rate of 120 K/h can be applied up to 250 °C (step 1). Beyond this point, the heating rate is reduced to 30 K/h (step 2). At this reduced heating rate, the furnace is heated to 640 °C and held at this temperature for 60 min to ensure the complete removal of residual binder.

Following this step, the furnace is purged with argon to remove residual hydrogen, and the atmosphere is shifted from hydrogen to argon (step 3). The temperature is then increased to the sintering temperature of 1110 °C at a rate of 120 K/h (step 4), which is then maintained for 90 min (step 5). After sintering, the furnace is gradually cooled to 400 °C. To accelerate the final cooling to ambient temperature, the upper section of the hinged tube furnace is opened (step 6). The entire sintering and cooling process is conducted under a controlled partial pressure of 300 mbar.

The sintering furnace enables a continuous flow of high-purity hydrogen gas (O_2_ < 2 ppm, H_2_O < 5 ppm) across the surfaces of the samples. Despite the gas purity, oxidation was observed at the sample edges. This effect is attributed to the continuous hydrogen flow during thermal debinding, which may introduce trace amounts of oxygen or moisture, leading to the oxidation of neodymium due to its high reactivity.

**Fig. 5:**
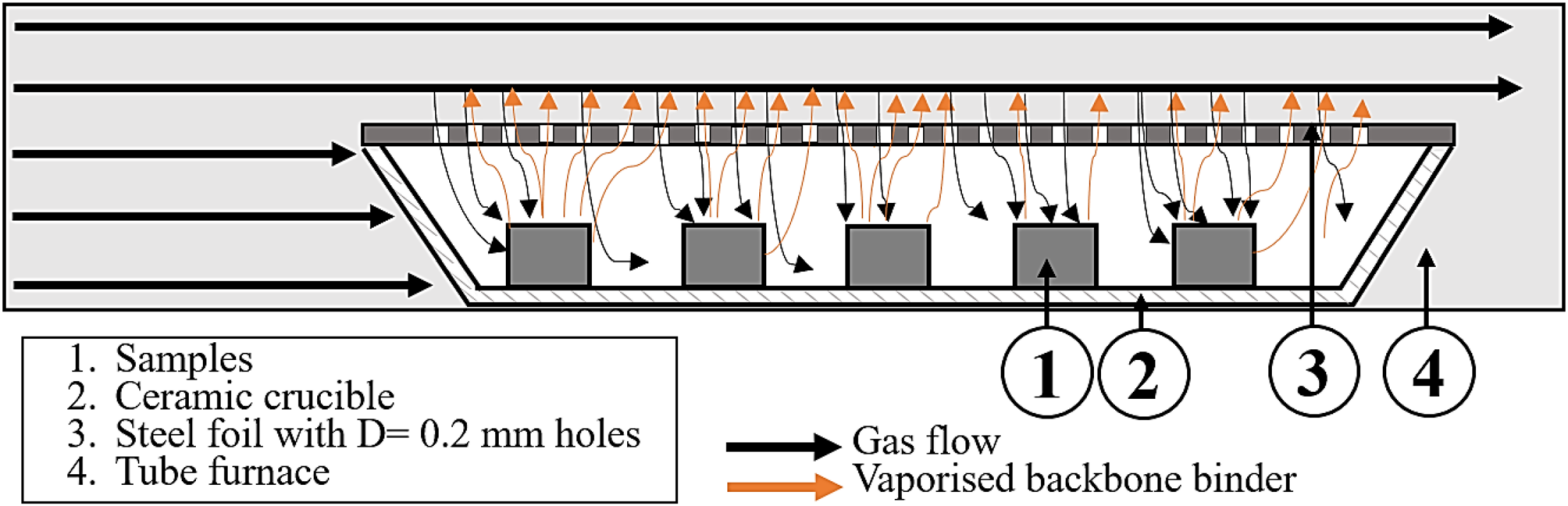
Schematic representation of the debinding and sintering process with covered samples.

To address this problem, the samples were covered with steel foil during the debinding and sintering processes. The foil functions as a physical barrier, effectively limiting the direct interaction between the sample surfaces and the surrounding gas atmosphere. Figure 5 illustrates a schematic of the experimental setup. The NdFeB samples (1) are placed in a ceramic crucible (2), which is partially covered with a steel foil (3). The black arrows in the schematic indicate the direction of the hydrogen gas flow during processing. To enable the release of vaporized backbone binder during thermal debinding, small holes with a diameter of 0.2 mm are drilled into the steel foil. These openings are essential; without them, the gaseous products of binder decomposition cannot escape, leading to pore formation and structural defects. The orange arrows illustrate the path of the escaping binder vapors.

Heat treatment is a post-sintering step to enhance the magnetic performance of NdFeB magnets. It promotes a more uniform distribution of the Nd-rich grain boundary phase, reduces structural defects, and thus improves grain isolation and microstructural stability. These effects increase coercivity, enhance the squareness of the demagnetization curve, and strengthen resistance against demagnetization^30,31^.

In the present study, heat treatment was carried out in two steps: 900 °C for 1 h under vacuum with subsequent cooling to room temperature, followed by 560 °C for 1 h under vacuum.

Since the Curie temperature is exceeded during sintering, the samples lose their magnetic order and require remagnetization. In this study, remagnetization was performed using a K-series pulse magnetizer (Magnet–Physik Dr. Steingroever GmbH, Cologne, Germany) by applying three consecutive 2000 V pulses along the easy axis.

### Analytical methodology

This section describes the analytical techniques used to characterize the magnetic, chemical, and microstructural properties of the samples.

From each magnet, cubic samples measuring 5 mm × 5 mm × 5 mm were extracted. Magnetic properties, including remanence *B*_*r*_, intrinsic coercive force *H*_*cj*_, and maximum energy product (*BH*_max_), were determined using a Hystograph HG 200 (Dr. Brockhaus Messtechnik GmbH & Co. KG, Lüdenscheid, Germany). Measurements were performed at room temperature, and corrections for demagnetization fields were applied using the standard Hystograph software based on the measured sample geometry and weight.

2 $$\:cos\phi\:=\frac{{B}_{r}}{{J}_{s}}$$

The quality of magnetic particle alignment can be assessed by determining the ratio between the remanent polarization *B*_*r*_ and the saturation polarization *J*_*s*_, as shown in Eq. 2.

In the case of fully aligned magnetic particles, this ratio approaches the value 1. A value of 0.5 indicates that the grains exhibit equal remanent polarization in all directions, i.e., the material is isotropic. This behaviour corresponds to the Stoner–Wohlfarth model for non-interacting, randomly oriented single-domain particles^32^. In exchange spring magnets, however, the remanence can exceed 0.5 due to magnetic coupling effects between grains, even when the easy axes are randomly distributed. All measurements were performed with the magnetic field applied parallel to the easy axis of magnetization.

The measurement principle relies on inductive detection of the magnetic flux in a sample placed between the pole pieces of an electromagnet. A sensing coil records the induced voltage, whose integration yields the magnetic flux. From the known sample dimensions, the flux density and polarization are calculated, enabling the determination of key magnetic parameters such as remanence, coercive field strengths, and maximum energy product.

The chemical composition of the magnets was analyzed using inductively coupled plasma optical emission spectroscopy (ICP–OES).

Oxygen and carbon contents were measured using an ONH 836 and a CS744 analyzer, respectively (both Leco Instrumente GmbH, Mönchengladbach, Germany).

The milled powder was analyzed using a Mastersizer 3000 particle size analyzer (Malvern Panalytical GmbH, Nuremberg, Germany) to determine its particle size distribution.

Microstructural analysis was performed using a Flex SEM 1000 scanning electron microscope (Hitachi High–Tech, Krefeld, Germany). Images were captured in backscattered electron (BSE) mode to reveal Nd-rich grain boundary phases and porosity.

## Results and discussion

### Chemical composition

The chemical compositions of the two end-of-life magnets and sintered PEM-magnets are compared in Table 2.

**Table 2:** Chemical composition eol magnets.

Magnet	Al	B	Co	Cu	Dy	Fe	Ga	Nd	Pr	Tb	RE total
M1 start	0.46	1.01	–	0.05	4.34	65.59	–	27.37	0.30	–	31.71
M1 PEM	0.42	1.01	–	0.03	4.11	65.44	–	29.51	0.12	–	33.74
M2 start	0.24	0.95	2.45	0.16	3.48	67.23	0.16	20.28	6.35	0.47	30.11
M2 PEM	0.23	0.94	1.66	0.12	3.39	66.74	0.13	24.11	4.22	0.31	32.02

Analysis of the chemical composition indicates that starting magnet M2 was deliberately Modified through the addition of 2.45 wt% cobalt (Co), a well-established alloying element in NdFeB permanent magnets known to enhance both coercivity *H*_*cj*_ and thermal stability^33–35^. Furthermore, 0.16 wt% Gallium (Ga) was detected in starting magnet M2. Huang et al.^36^ reported that adding 0.3–0.5 wt% Ga to NdFeB magnets significantly increases coercivity by improving the wettability of grain boundary phases, leading to smooth, continuous, and thin boundary layers that enhance magnetic decoupling between grains.

Heavy rare earths (HRE) such as dysprosium (Dy) and terbium (Tb) are often added to increase coercivity and thermal stability by replacing Nd atoms in the Nd_2_Fe_14_B lattice. Owing to their higher magnetocrystalline anisotropy constant compared to Nd, HREs enhance resistance to magnetization reversal, thereby improving *H*_*cj*_. However, excessive Dy addition can lower remanence *B*_*r*_, since Dy magnetic moments couple antiparallel to those of the Fe sublattice, reducing saturation magnetization^35,37^.

In both starting magnets, the total HRE content is about 4 wt%. Chemical composition analysis shows that M1 contains slightly more HREs (4.34 wt%) than M2 (3.95 wt%). In M1, the HRE fraction consists entirely of Dy, whereas in M2 it is composed of Dy (3.48 wt%) and Tb (0.47 wt%). Starting magnet M2 also contains substantially less Nd (20.28 wt%) than M1 (27.37 wt%), with the deficit largely compensated by a higher Pr content of 6.35 wt% (M1: 0.3 wt%).

The partial substitution of Nd with praseodymium (Pr) in NdFeB magnets provides an economic advantage, as Pr is more abundant and less expensive than dysprosium. At the same time, Pr supports the development of high coercivity, allowing a partial replacement of Dy-containing alloys. However, increasing Pr content slightly decreases the remanence *B*_*r*_, since the saturation magnetization of Pr_2_Fe_14_B is lower than that of Nd_2_Fe_14_B. Although Pr couples ferromagnetically with the Fe sublattice, its smaller number of unpaired 4f electrons results in a reduced magnetic moment compared to Nd^37–39^.

After recycling, both magnet types M1 and M2 exhibit an increase of approximately 2 wt% in Nd and in the total rare earth RE content, resulting from the addition of NdH_~2.7_. This relative increase is accompanied by a slight decrease in Dy and Pr contents, as well as a small reduction in Fe. The latter is mainly due to the higher proportion of rare earths, which causes a corresponding decrease in the relative fractions of the remaining alloying elements.

### Oxygen and carbon content of the sintered PEM-samples

**Fig. 6:**
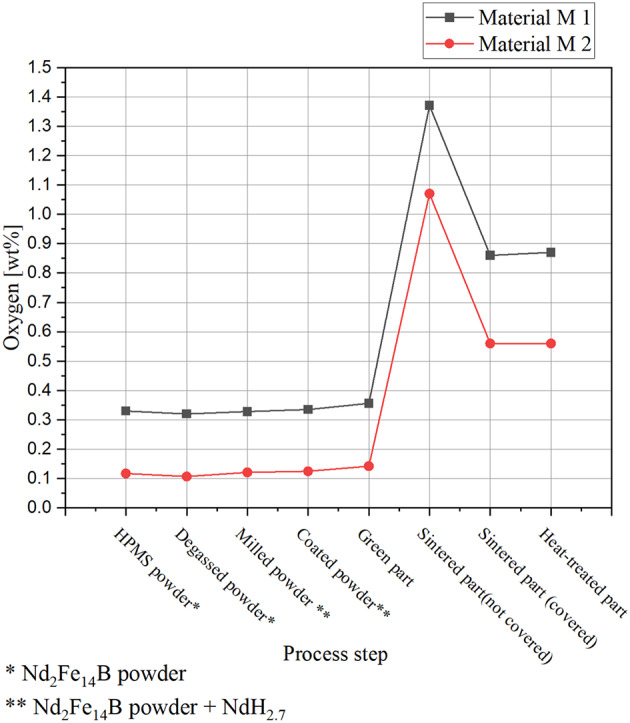
Oxygen content, measured after the process steps.

In the initial processing steps, including powder production and the extrusion of the green parts, the oxygen content remains relatively stable for both materials, with no significant oxygen uptake observed during degassing, milling, or shaping, Fig. 6. However, the initial oxygen content of material M1 is notably higher at 0.33 wt% compared to 0.12 wt% for material M2. Even during feedstock preparation and extrusion, only a minimal increase in oxygen content was detected, with values remaining around 0.1 wt%, indicating that these stages contribute negligibly to overall oxygen incorporation.

However, a pronounced increase in oxygen content is observed after the sintering step. For material M1, the sintered PEM sample without a protective cover exhibited an oxygen content of 1.37 wt%, whereas the application of a protective cover during sintering effectively reduced this value to 0.87 wt%. A similar trend was observed for material M2: the uncovered sample reached an oxygen level of 1.07 wt%, which decreased to 0.56 wt% when a protective cover was used during the sintering process, Fig. 5.

The measurements of oxygen content after the individual processing steps indicate that oxygen uptake remains below the critical threshold of 1.0 wt%, beyond which magnetic properties are significantly degraded^8^. The results suggest that an overall increase in oxygen content of approximately 0.45–0.5 wt% can be expected throughout the production process. Moreover, the data highlight the importance of starting material quality in the manufacturing of recycled magnets. Higher initial oxygen levels narrow the process window for producing NdFeB permanent magnets with favourable magnetic properties via the PEM process. It is therefore imperative to protect the process from oxygen contamination to minimise the amount of oxygen that can enter the structure, as the oxygen content can vary when using recycled material.

**Fig. 7:**
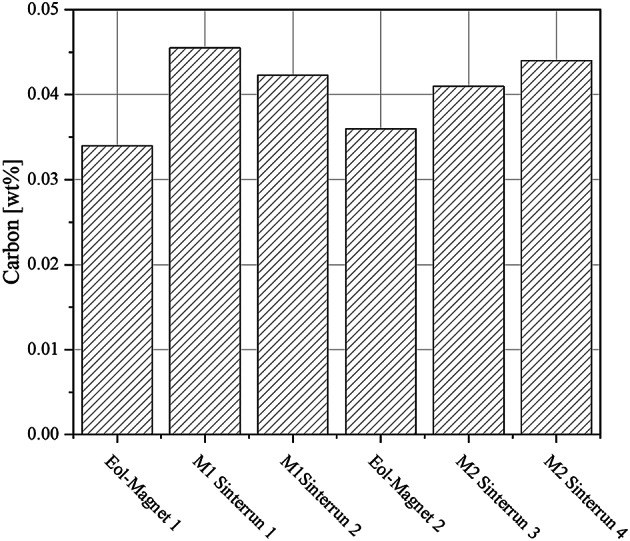
Carbon content of Eol-magnets and sintered PEM-samples.

The carbon content of sintered PEM magnets produced from two starting materials, M1 and M2, across four separate sintering runs under identical conditions is shown in Fig. 7. The results demonstrate relatively consistent carbon levels among all samples, with values ranging from approximately 0.041 wt% to 0.046 wt%. For material M1, the first sintering run shows the highest carbon content, approximately 0.046 wt%, followed by a slight decrease in the second run. In the case of material M2, the lowest carbon content is observed in sintering run 3 with approximately 0.041 wt%, with a marginal increase in run 4. The carbon content of the initial magnets is low, with M1 at 0.034 wt% and M2 at 0.036 wt%, indicating only minor carbon uptake.

All samples underwent debinding at a heating rate of 30 K/h in a hydrogen atmosphere, as described in Figs. 4 and 5. There is close agreement with the results of Lopes et al.^19^ and Hartwig et al.^9^ who also determined values of approximately 0.05 wt% carbon at a heating rate of 30 K/h in hydrogen.

Overall, the minor variations between sintering runs indicate good reproducibility of the debinding and sintering process with respect to carbon removal. The consistently low carbon contents suggest that both the binder system and the applied process parameters are effective in minimizing carbon residues in the final PEM magnets.

### Microstructural analysis

**Fig. 8:**
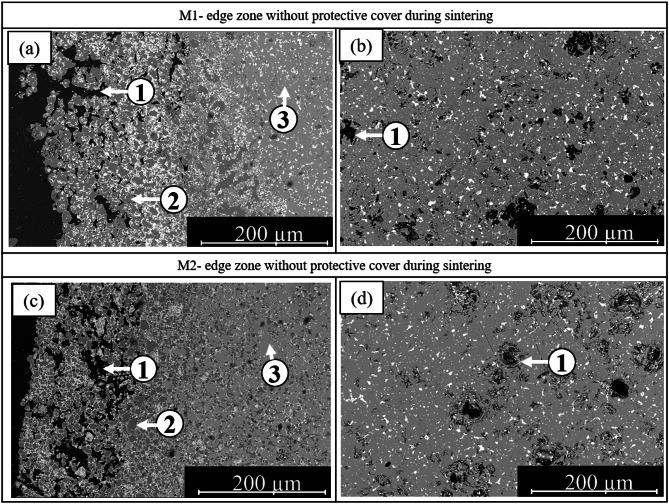
(**a**) Edge zone of PEM magnet M1 and M2 without protective cover during sintering; (**b**) middle zone of PEM magnet M1 without protective cover. (1) Pores, (2) α-Fe, (3) Nd_2_Fe_14_B phase.

SEM images of the microstructures of sintered NdFeB magnets without a protective steel cover during the sintering process are shown in Fig. 8. The images allow for a comparative analysis of the edge and center regions of the samples. All samples were prepared using the debinding and sintering process outlined in Figs. 4 and 5.

Pores (1) of various sizes are visible up to approximately 150 μm from the edge in the edge and center regions of sample M1–160 °C (Fig. 8a, b). Dark gray areas (2) correspond to α-Fe. Between 150 μm and 250 μm from the edge, the microstructure becomes denser, although isolated α-Fe regions are still present. Closer to the center, a compact Nd_2_Fe_14_B (3) microstructure is observed, but pores of varying sizes remain, although no α-Fe phases are detected.

In the M2 sample, the microstructure is similar, Fig. 8c, d. The edge region is clearly oxidized and contains larger pores, with α-Fe phases distinctly visible. In the further course to the middle region, no α-Fe is recognizable either.

**Fig. 9:**
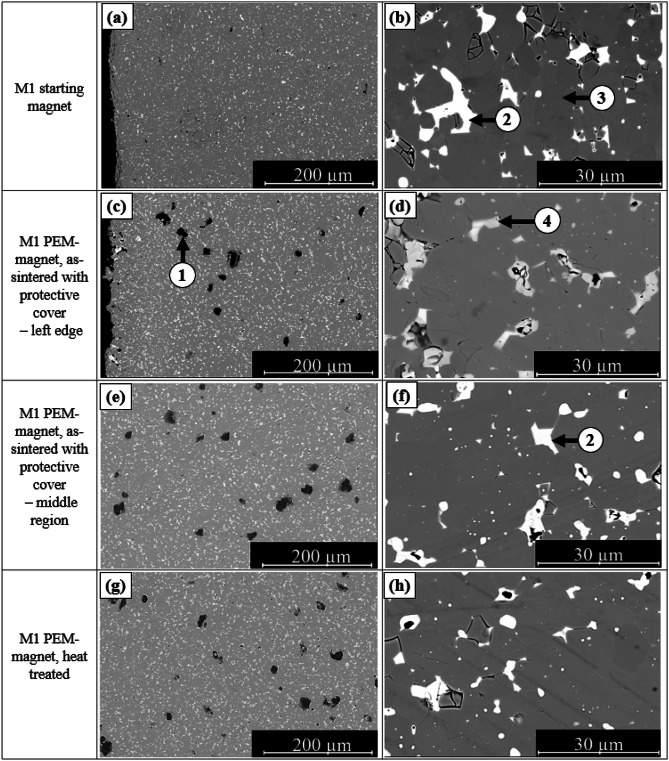
SEM images of M1. (**a**, **b**) Starting magnet; (**c**, **d**) PEM magnet in as-sintered state (with protective cover during sintering); (**e**, **f**) PEM magnet in as-sintered state (without protective cover during sintering); (**g**, **h**) PEM magnet after heat treatment. (1) Pores, (2) Larger Nd-agglomerates, (3) Nd_2_Fe_14_B phase, (4) oxidized Nd-rich phase.

**Fig. 10:**
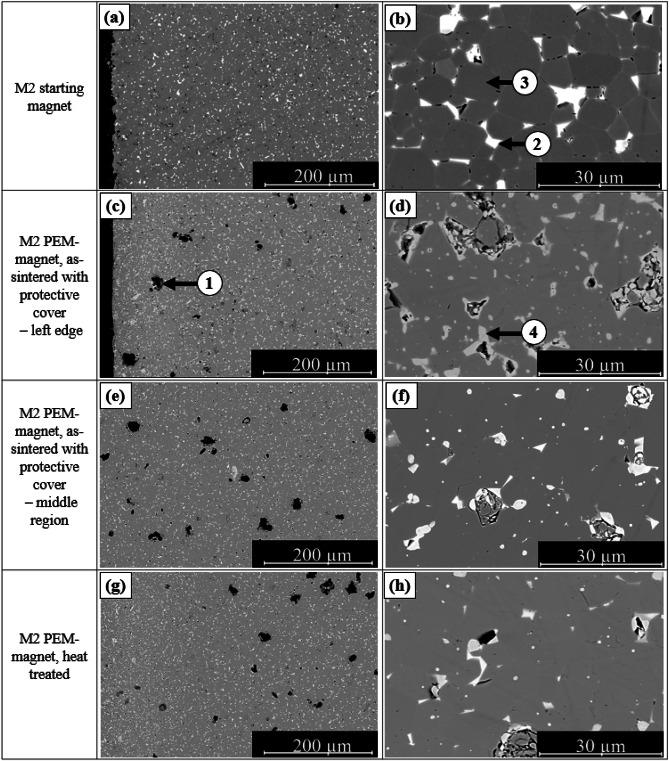
SEM images of M2. (**a**, **b**) Starting magnet; (**c**, **d**) PEM magnet in as-sintered state (with protective cover during sintering); (**e**, **f**) PEM magnet in as-sintered state (without protective cover during sintering); (**g**, **h**) PEM magnet after heat treatment. (1) Pores, (2) Larger Nd-agglomerates, (3) Nd_2_Fe_14_B phase, (4) oxidized Nd-rich phase.

The microstructures of the starting magnets, the sintered PEM magnets, and the heat-treated PEM magnets M1 and M2 are shown in Figs. 9 and 10. Samples M1-200 °C and M2-200 °C are presented, as they exhibited the best magnetic properties. The initial magnets display dense, nearly pore-free microstructures with a homogeneously distributed Nd-rich phase (light contrast) in both materials (Figs. 9a and b and 10a and b). In M2, the Nd-rich phase is more continuous along the grain boundaries, enhancing grain separation and likely contributing to its higher coercive field strength.

Compared to uncovered PEM magnets, the covered samples exhibit a denser and more homogeneous microstructure, free of α-Fe and with significantly fewer pores. The edge zone contains no α-Fe, and pores between 5 μm and 20 μm are evenly distributed across the cross-section (Figs. 9c, d and 10c, d). These pores may originate from residual gas inclusions due to incomplete removal of vaporized binder during debinding, or from sample preparation, where oxidized NdH_~2.7_ particles detach during polishing and leave voids. At the sample edges, darker Nd-rich phases are visible, indicating oxidation of the Nd-rich phase.,

The comparison of sintered and heat-treated samples shows no significant changes in the microstructure (Figs. 9e, f and 10e, f). In both conditions, the samples of both materials display a dense microstructure with finely distributed Nd-rich phases along the grain boundaries.

### Magnetic properties of green parts and sintered parts

The influence of nozzle temperature on the magnetic alignment and performance of extruded green parts and sintered PEM magnets produced from the two different starting materials, M1 and M2, is shown in Fig. 12. A temperature sensor is used to measure the temperature on the inner surface of the nozzle. The diagrams illustrate the temperature-dependent development of the alignment quality and magnetic properties.

The *B*_*r*_*/J*_*s*_ ratio of the green parts as a function of nozzle temperature, serving as an indicator of the degree of magnetic alignment, is shown in Fig. 12a. For green parts from material M1, the degree of alignment increases steadily with rising nozzle temperature, from 0.67 at 160 °C to 0.84 at 200 °C. A similar trend is observed for green parts from material M2, where the *B*_*r*_*/J*_*s*_ ratio rises from 0.69 at 160 °C to approximately 0.84–0.85 at 200 °C. Both materials exhibit a pronounced increase between 170 °C and 180 °C, suggesting a threshold temperature range where particle orientation is significantly enhanced during extrusion.

Provided the material exhibits sufficient coercivity and a uniform microstructure, the remanence *B*_*r*_, and hence *BH*_max_, is governed by three factors: (i) the intrinsic saturation magnetization of the magnetic phase (affected only by composition), (ii) the volume fraction of the magnetic phase, which can be increased by higher density and a lower fraction of non-magnetic secondary phases or binder, and (iii) the degree of crystallographic alignment in anisotropic magnets, with the maximum reached when the c-axis of each grain is oriented along the magnetization direction^40^.

The remanence *B*_*r*_ of the starting magnet M1 was measured at 1200 mT, whereas M2 exhibited a slightly higher value of 1261 mT, Table 3. The different remanence values of the starting magnets M1 and M2 can primarily be attributed to differences in the chemical composition and the degree of alignment of the magnetic particles. At 67.23 wt%, M2 has an Fe content that is about 1.6 wt% higher than M1 (65.59 wt%), which leads to a slight increase in the saturation polarization *J*_*s*_. The total rare earth content is slightly lower in M2 (30.11 wt%) than in M1 (31.71 wt%), while the proportion of heavy rare earth metals (Dy + Tb) is comparable in both magnets, so that no significant contribution of these elements to the observed difference in remanence *B*_*r*_ is to be expected. The addition of Pr does not have a negative effect on the saturation of the starting magnet M2, as the *J*_*s*_ values are almost identical to those of M1. The higher *B*_*r*_*/J*_*s*_ value of 0.99 for M2 compared to 0.96 for M1 suggests that the higher remanence of M2 is primarily due to its better crystallographic orientation.

The measured values of *J*_*s*_ depend not only on the intrinsic properties of Nd_2_Fe_14_B but also on the degree of grain alignment. Due to the high magnetocrystalline anisotropy of Nd_2_Fe_14_B, magnetic moments preferentially align along the crystallographic c-axis. If this axis is inclined relative to the measurement direction, only the projection of the magnetic moment is detected, leading to an apparently reduced *J*_*s*_. The hystograph outputs this measured value as *J*_*s*_; in misaligned samples it is lowered by projection effects, although the intrinsic *Js* of the Nd_2_Fe_14_B phase remains unchanged.

A clear increase in remanence *B*_*r*_ and saturation polarization *J*_*s*_ is observed with rising nozzle temperature (Fig. 12c and Table 3). For the samples produced from material M1, remanence *B*_*r*_ increases from approximately 750 mT at a nozzle temperature of 160 °C to 984 mT at 200 °C. Compared to the Eol magnet, the sintered PEM magnet M1–200 °C exhibits a reduced *B*_*r*_ of 984 mT, corresponding to approximately 82% of the *B*_*r*_ value of the Eol magnet, while *J*_*s*_ is also lower, at 1027 mT. Samples made from material M2 start at a lower *B*_*r*_ of 684 mT and reach a maximum of around 882 mT at a nozzle temperature of 200 °C. The observed increase in *B*_*r*_ and *J*_*s*_ with improved processing temperature is consistent with enhanced grain alignment, which reduces projection losses and enables measurement values closer to the intrinsic saturation polarization of the material to be obtained.

When measuring magnets with a hystograph, non-magnetic pores or inclusions reduce the effective cross-section of the sample and disrupt the magnetic flux path. Consequently, the measured saturation polarization appears lower due to volume normalization, while local field inhomogeneities may further decrease the absolute saturation value. The low *J*_*s*_ of M1–200 °C (1027 mT) compared with the starting magnet is attributed to microstructural and compositional defects, while the degree of alignment is the same for both (0.96). In addition to the higher Nd content, the presence of larger Nd-rich agglomerates and an oxygen uptake of about 0.4 wt%, leading to non-magnetic oxide phases, reduce *J*_*s*_. The lower density (7.1–7.2 g/cm³) and visible porosity further decrease the magnetically active volume, compounding the observed loss in *J*_*s*_.

The recycled PEM magnet from material M2 shows a saturation polarization *J*_*s*_ of only 963 mT compared to 1260 mT for the corresponding Eol-magnet, with the alignment degree decreasing from 0.99 to 0.92. Since the green parts were as well aligned as in magnet M1, the magnetic particles in the samples of M2 lose some of their alignment during thermal debinding and sintering. The exact cause of this loss of orientation is not yet clear. It should therefore be addressed in the discussion that further debinding and sintering experiments are required to determine the optimal sintering parameters for this material, in order to minimize orientation loss and maximize magnetic performance. A minor influence on the lower magnetization could be the content of Pr in M2. Pr couple ferromagnetically with the Fe sublattice, but contribute a slightly smaller magnetic Moment than Nd due to the lower number of unpaired 4f electrons. In material M2, the Pr content (4.22 wt%) is higher and the Nd content (24.11 wt%) is lower than in M1 (0.3 wt% Pr, 27.37 wt% Nd), which could lead to a minor reduction in saturation polarization *J*_*s*_. However, it remains unclear whether this effect significantly contributes to the lower *J*_*s*_ observed in M2, and its influence relative to Fe content and degree of alignment cannot be confirmed from the present data.

**Fig. 11:**
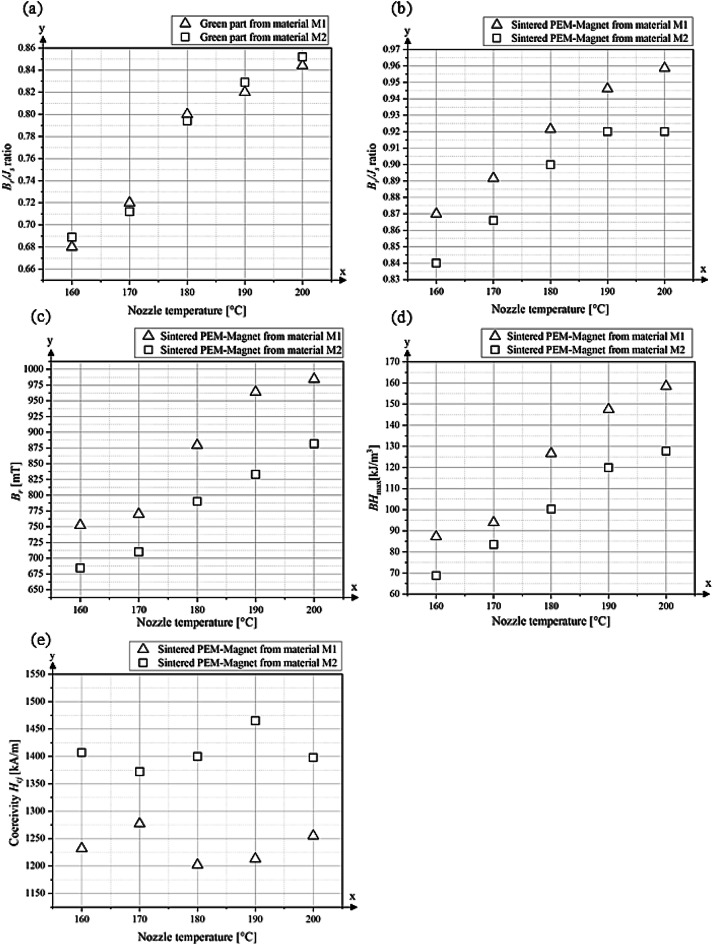
*B*_*r*_*/J*_*s*_ ratio of (**a**) green part (**b**) *B*_*r*_*/J*_*s*_ ratio sintered part, (**c**) Remanence *B*_*r*_ sintered part, (**d**) *BH*_max_ sintered part, (**e**) Coercivity *H*_*cj*_.

**Fig. 12:**
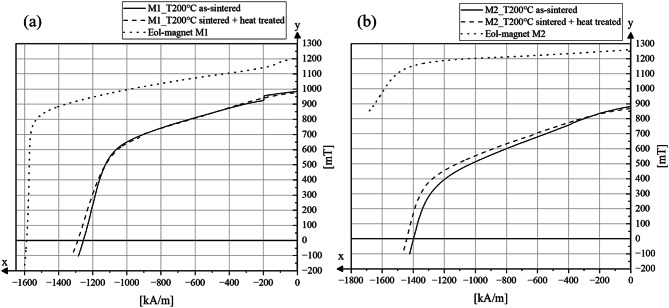
Demagnetization curves of sintered and heat-treaded samples for magnet 1 and magnet 2.

The energy product *BH*_max_, increases in accordance with the higher remanence *B*_*r*_ values as the nozzle temperature rises (Fig. 12d). For material M1, the maximum *BH*_max_ reaches approximately 158 kJ/m^3^ at a nozzle temperature of 200 °C. In comparison, material M2 achieves a slightly lower peak value of around 135 kJ/m^3^ at the same temperature. The measurements show that *B*_*r*_*/J*_*s*_, *B*_*r*_ and *BH*_max_ increase systematically with nozzle temperature for both M1 and M2 (Fig. 11). According to the magneto–rheological model by Jung et al.^12^, this can be explained by a reduction in binder viscosity at elevated temperatures, which lowers the Mason number. A lower Mason number means that magnetic torque dominates over hydrodynamic drag, allowing particles to align more effectively with the applied magnetic field before solidification.

The coercivity *H*_*cj*_ of the sintered PEM magnets produced from material M1 lies between around 1215 kA/m and 1277 kA/m and shows no clear dependence on the nozzle temperature. The values for material M2 are significantly higher than for the starting magnet M1, between 1372 kA/m and 1465 kA/m. The starting material of M2 was optimized through targeted alloying to increase coercivity (Sect. 3.1), which is also reflected in the values for coercivity in the recycled PEM samples. Compared to the initial magnet, however, the coercivity of both PEM magnets is significantly lower. Micrographs of the sintered PEM samples reveal larger pores distributed within the microstructure Figs. 9 and 10. These pores act as nucleation sites for reversed magnetic domains, where the local magnetic reversal is initiated at lower applied field. During thermal debinding and sintering, the samples absorb oxygen, as shown in Fig. 6. The Nd-rich grain boundary phase is particularly prone to oxidation, and oxidized regions at the grain boundaries can be clearly observed. Oxidation transforms this phase into non-functional oxides, impairing its role as a non-magnetic boundary layer that isolates the hard magnetic Nd_2_Fe_14_B grains. This loss of magnetic isolation promotes intergrain coupling and leads to a decrease in coercivity.

All magnetic values of the starting magnets and the sintered PEM magnets are shown in Table 3.

**Table 3:** Magnetic values and density of sintered samples.

Sample	Coercivity H_cj_ [kA/m]	Remanence B_*r*_ [mT]	Saturation J_s_ [mT]	BH_max_ [kJ/m3]	B_*r*_/J_s_ ratio II easy-axis	Density [g/cm^3^]
Starting magnet M1	1592	1200	1253	240	0.96	7.47
Starting magnet M2	> 1778	1261	1267	300	0.99	7.50
M1–T160°C	1232	752	863	87	0.87	7.22
M1–T170°C	1277	783	869	97	0.90	7.21
M1–T180°C	1202	879	954	127	0.92	7.19
M1–T190°C	1213	964	1019	148	0.95	7.20
M1–T200°C	1255	984	1027	158	0.96	7.21
M2–T160°C	1407	684	817	69	0.84	7.21
M2–T170°C	1372	710	822	86	0.86	7.20
M2–T180°C	1400	790	878	100	0.90	7.19
M2–T190°C	1465	833	905	120	0.92	7.16
M2–T200°C	1398	882	963	128	0.92	7.21

The degree of crystallographic alignment in sintered NdFeB magnets is strongly influenced by the particle size of the starting powder. Both excessively fine and overly coarse powders reduce the resulting remanent magnetization *B*_*r*_. Fine powders tend to agglomerate due to strong interparticle magnetic interactions, hindering effective alignment during magnetic field-assisted compaction, and their high surface energy promotes oxidation. This oxidation reduces the fraction of magnetic Nd_2_Fe_14_B phase, forms non-magnetic phases such as α–Fe, and can increase porosity. In addition, fine particles accelerate grain growth during sintering, potentially degrading magnetic texture. Conversely, coarse powders lead to a heterogeneous microstructure with poor grain boundary contact, weakening magnetic exchange coupling. Sun et al. [42] reported that a particle size of about 4.9 μm yields the highest alignment, while larger particles show lower alignability. In the present study, the milled powder had D_v_(10), D_v_(50), and D_v_(90) values of 2.97 μm, 7.68 μm, and 16.1 μm, respectively, indicating a relatively coarse size distribution that likely limits alignment and impairs the final magnetic properties.

The demagnetization curves of the two Eol magnets and the corresponding PEM samples, both in the as-sintered state and after heat treatment, are shown in Fig. 12. Among the extruded samples, those processed with a melt temperature of 200 °C demonstrated the most favorable magnetic properties and were therefore selected for subsequent heat treatment. The heat treatment process, as described in Sect. 2, was conducted in two stages. In the first step, the samples were heated to 900 °C and then cooled to room temperature. In the second step, the samples were reheated to 560 °C. Both stages were performed under vacuum conditions, with a holding time of 1 h at each target temperature.

The demagnetization curves of the samples produced from material M1 are shown in Fig. 12a. Eol magnet M1 exhibits a remanence *B*_*r*_ of approximately 1200 mT and a coercivity *H*_*cj*_ of around 1592 kA/m. The shape of the demagnetization curve shows a slightly reduced squareness, which could indicate a non-ideal microstructure characterized by a magnetic decoupling of the individual grains. This behavior implies limited or disrupted exchange coupling between the Nd_2_Fe_14_B grains. Such decoupling is often associated with the presence of intergranular defects or non-magnetic boundary phases. These defects can be seen in Fig. 9c–f. This interpretation is further supported by the elevated oxygen content measured in the starting material (Fig. 6), which can lead to the formation of Nd-oxides at grain boundaries and hinder magnetic exchange interactions. Another reason for this is the non-perfect degree of alignment of 0.96. This can also lead to a reduction in squareness^41^.

Compared to the Eol reference magnet, the demagnetization curve of the PEM magnet 1 shows a further deformation of the rectangularity. This behavior can primarily be attributed to the presence of pores in the microstructure, which act as pinning centers and locally hinder the reversal of the magnetic domains. As a result, higher external fields are required to overcome these local energy barriers, leading to a more gradual decrease in magnetization and a less square hysteresis. Furthermore, the comparison with the heat-treated sample reveals no significant change in coercivity *H*_*cj*_ or the angular shape of the demagnetization curve, indicating that the thermal post-treatment does not substantially alter the magnetic reversal behavior.

The demagnetization curves show remarkable differences in the magnetic behavior of Eol magnet M2 compared to the PEM samples (as-sintered and heat-treated) from material M2, as shown in Fig. 12b. Eol magnet M2 shows a remanence of about 1260 mT, together with a nearly horizontal course of the curve. It should be noted that for Eol magnet M2, coercivity *H*_*cj*_ could no longer be measured accurately at room temperature, indicating an exceptionally high coercivity beyond the accessible measurement range. The measurement was aborted at a value of 1778 kA/m.

In comparison, the demagnetization curves of PEM magnets show a slightly steeper decline than the Eol reference magnet. The remanence is significantly lower at around 880 mT, which corresponds to almost 70% of the reference value for *B*_*r*_ of Eol magnet M2. The shape of the demagnetization curve of the sintered and heat-treated PEM sample shows a significantly lower rectangularity than the initial magnet and thus a gradual reversal of the magnetic domains. This is also due to the structural defects, but also to the lower degree of alignment. The heat treatment leads to a minimal improvement in the coercivity *H*_*cj*_ and the curve shape, which may indicate a reduction in defects or stresses. However, the heat treatment does not have a major effect here either.

## Summary/further work

In this study, the individual processing steps for the production of anisotropic NdFeB permanent magnets from recycled material were systematically investigated. Two Eol magnets served as the starting materials and were converted into powder using the HPMS method. The process chain was analysed with particular focus on the oxygen uptake, from HPMS powder through to the sintered and heat-treated PEM magnets.

The results indicate that most oxygen uptake occurs during thermal debinding and sintering. Future work should investigate sintering under high-vacuum conditions as an alternative to the current partial-pressure setup and assess whether finer powder fractions in PEM feedstocks can be used without exceeding acceptable oxygen levels. For material M2 containing Pr, additional debinding and sintering trials are needed to clarify the cause of grain reorientation.

The carbon content in the final MIM/PEM magnets remained low, between 0.04 wt% and 0.05 wt%, indicating that the debinding strategy employed is effective in removing organic binder residues.

The results of the magnetic measurements show that it is possible to produce an anisotropic NdFeB magnet with a degree of alignment of 0.96. The extrusion tests carried out at different melting temperatures showed that the optimum conditions for achieving the maximum degree of alignment are at temperatures between 190 °C and 200 °C. To achieve further improvement in remanence *B*_*r*_ and coercivity *H*_*cj*_, optimization of the microstructure of the samples is essential. The reduction of the magnetic phase caused by the pores in the microstructure leads to a decrease in remanence. In addition, pores act as pinning centres, leading to a less rectangular demagnetization curve and thus a reduction in coercivity.

For further optimization of the PEM process in the production of high-quality NdFeB permanent magnets, the use of finer powder is essential. The results of this study demonstrate that particularly coarse NdH_~2.7_ particles contribute to the formation of defects within the microstructure, which adversely affect the magnetic properties, most notably the coercivity *H*_*cj*_. Moreover, the use of finer powder may improve the alignment of the magnetic particles during shaping, thereby enhancing the degree of magnetic alignment and remanence in the final magnet.

## Supplementary Information

Below is the link to the electronic supplementary material.

Supplementary Material 1

## Data Availability

All data supporting the findings of this study are available within this article.
