# Supplementary material for: Process optimization to produce anisotropic NdFeB permanent magnets from recycled powder via powder extrusion
Source: Sci Rep. 2025 Oct 9;15:35284. doi: 10.1038/s41598-025-19111-6 (PMC12511325; doi:10.1038/s41598-025-19111-6)
Supplement: Supplementary file 1 — Supplementary Material 1 [file 41598_2025_19111_MOESM1_ESM.pdf]

Supplementary Material

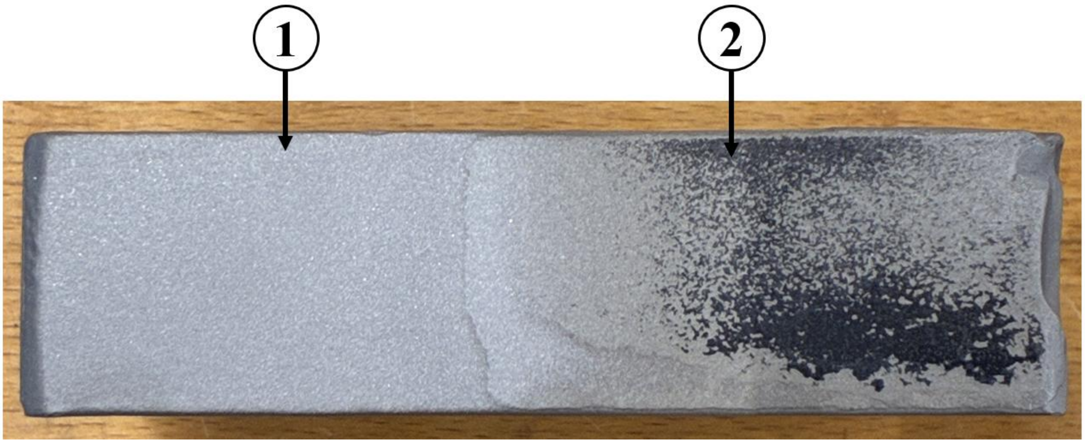

Supplementary Figure A1. Epoxy-coated NdFeB magnet: (1) sandblasted surface; (2) surface with coating
